# The unusual experience of managing a severe COVID-19 case at home: what can we do and where do we go?

**DOI:** 10.1186/s12879-020-05608-0

**Published:** 2020-11-19

**Authors:** Ivan Chérrez-Ojeda, Emanuel Vanegas, Miguel Felix

**Affiliations:** 1 Universidad Espíritu Santo, Km. 2.5 vía La Puntilla, 0901-952 Samborondón, Ecuador; 2 Respiralab Research Group, Guayaquil, Ecuador

**Keywords:** Ambulatory care, COVID-19, Developing countries, South America

## Abstract

**Background:**

The speed and reach of the COVID-19 pandemic has created special scenarios to be considered, such as those in where patients who meet criteria for hospitalization due to moderate/severe disease cannot be hospitalized due to economic constraints and saturation of national health systems. The aim of this report is to present an unusual case of a severe COVID-19 patient managed at home in a developing country, and to discuss some of the available guidelines and potential therapeutic options for this type of cases.

**Case presentation:**

A 60-year-old female seeking medical attention through teleconsultation presents with profound dyspnea, oppressive chest pain, fatigue, episodic hallucinations, and difficulty sleeping, for what she originally sought medical attention at an ER but could not be admitted due to saturation of the health system. A positive PCR test for COVID-19, and a CT scan of the chest showing bilateral consolidations with ground-glass opacities confirmed the diagnosis. The patient was managed at home, with corticosteroids, nitazoxanide and a single dose of 40 mg of subcutaneous enoxaparin. Colchicine was added at the third day of treatment. Standard oxygen therapy through nasal cannula was also recommended. Daily follow-ups were established to monitor for signs of clinical improvement. Two weeks later from the initial consultation the patient presents marked improvement in her symptoms, as well as in her CT scan, which prompted in discontinuation of the medications and the oxygen therapy.

**Conclusions:**

There are several limitations in this report regarding the clinical data and the management, but such limitations do also reflect the state of emergency and the chaos that resides in the health care systems of developing nations. For the ambulatory care of COVID-19 patients, several aspects of disease management may differ from current guidelines and basic requirements may represent a huge challenge to cover. Further research is needed to assist physicians in the daily clinical decision making, to optimize patient outcomes, and to reduce the probability of adverse scenarios of patients with COVID-19 managed in the ambulatory setting.

## Background

To date, the COVID-19 pandemic has affected over 184 countries, representing a global health problem of enormous proportions not only as a health crisis, but one with devastating social and economic implications for years to come [1, 2]. Adding to the already complex scenario are the different responses to the pandemic between countries according to their own strengths and weaknesses, exposing a hard truth: inequality is a threat that will disproportionally hit developing countries [3]. According to a recent report by the World Economic Forum, developing countries suffer from a severe shortage of healthcare workers, as well as a lack of fiscal and monetary capacity to cope with the speed of the pandemic [4]. This in turn leads to a scenario where poverty, overcrowding, and poor public health systems, in combination with the virus, can affect individuals in practically any socioeconomic status and in any society [5].

The spectrum of symptomatic COVID-19 infection ranges from mild to critical, with roughly 80% of symptomatic patients presenting with mild disease [6]. Studies have found that older patients, with associated comorbidities such as cardiovascular disease, type 2 diabetes, chronic kidney and lung disease, and cancer, are at higher risk for severe and life-threatening disease [6–9]. In the case of severe patients, there are several agents with demonstrated in vitro activity against COVID-19 used in clinical practice, including hydroxychloroquine, remdesivir, interleukin-6 pathway inhibitors, and convalescent plasma; however, to date few agents have proven efficacy against COVID-19 [10]. For instance, a recent preliminary report on the antiviral medication remdesivir, found that it was superior to placebo in shortening the time to recovery among hospitalized adults with COVID-19 with evidence of lower respiratory tract infection, although a reduced mortality benefit was not seen [11]. On the other hand, ambulatory care is usually restricted to patients with asymptomatic to mild disease, and the management is based infection control, and symptomatic relief (e.g. antipyretic medications, and hydration) [12, 13].

However, the speed and reach of the pandemic has created special scenarios to be considered, such as those in where patients who meet criteria for hospitalization due to moderate/severe disease cannot be hospitalized due to economic constraints and saturation of national health systems. The aim of this report is to present an unusual case of a severe COVID-19 patient managed at home in a developing country, and to discuss some of the available guidelines and potential therapeutic options for this type of cases.

## Case presentation

A previously healthy 60-year-old female seeking medical attention through teleconsultation presents with profound dyspnea, oppressive chest pain, fatigue, episodic hallucinations, and difficulty sleeping. The patient describes that her symptoms began 7 days ago with non-productive cough and fever, later progressing to dyspnea that exacerbated with minimal effort, for what she originally sought medical attention at an ER. At the ER triage the patient presented with profound dyspnea, an oxygen saturation of 86% at room air, and a respiratory rate of 32 breaths/min. However, despite the severity of the respiratory compromise, the patient could not be admitted due to hospital oversaturation. Unfortunately, the patient was sent home with medical recommendations, including nebulization with normal saline, acetylcysteine and ambroxol hydrochloride. She was also advised that a chest CT scan was needed.

**Fig. 1:**
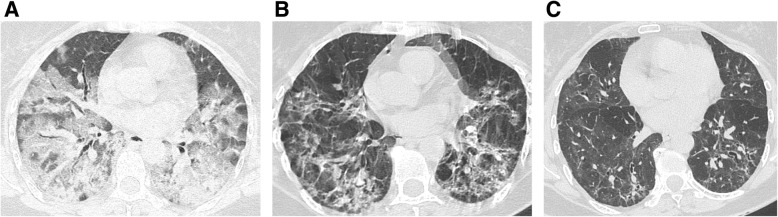
Computed tomography (CT) scans of the chest. **a** Initial CT scan showing bilateral ground glass opacities involving most of the lung parenchyma. **b** Follow-up CT scan 14 days later showing marked improvement from baseline. **c** Follow-up CT scan at 6 months

**Fig. 2:**
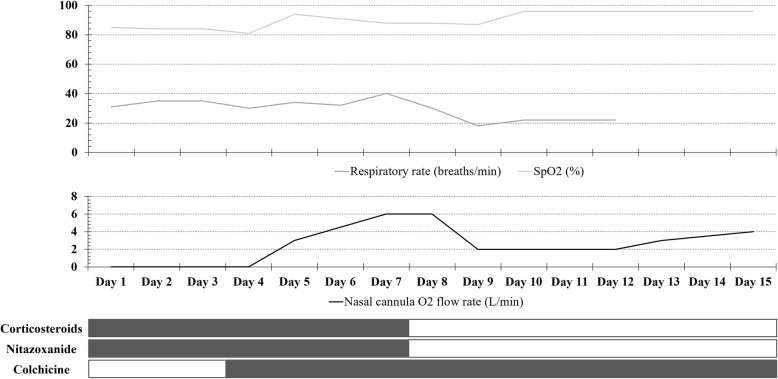
Timeline of the clinical parameters, oxygen requirement, and management of the patient

At the medical teleconsultation, the patient presented marked respiratory distress accompanied with a respiratory rate of 31 respirations per minute, and an oxygen saturation of 85% at room air. A relative of the patient described what is compatible with skin and mucous membrane dryness, skin pallor and bilateral 1+ pitting edema. The patient handed-off a copy of the CT scan where bilateral pulmonary edema and consolidations with ground-glassing were observed encompassing nearly 90% of the lung parenchyma (Fig. 1a). A basic panel of laboratory tests, and a PCR test for COVID-19 were ordered, the latter of which confirmed the diagnosis (Table 1). She was prescribed with IV normal saline for rehydration, pharmacologic management with IV methylprednisolone (250 mg every 24 h for 3 days), followed by prednisone (40 mg for 7 days), nitazoxanide (500 mg every 8 h) for 7 days and a single dose of 40 mg of subcutaneous enoxaparin, all of which were administered by an outpatient registered nurse at home. Standard oxygen therapy through nasal cannula was also recommended. Medical treatment is best depicted in Fig. 2. Colchicine was added at the third day of treatment (2 mg loading dose, followed by 0.5 mg every 8 h up to the end of treatment).

**Table 1:** Laboratory parameters at baseline evaluation

Parameter	Result	Reference Range
RBC × 10^3^	4.59	3.8–6
WBC × 10^3^	**21.38**	3.90–10.80
Neutrophils × 10^3^	**18.77**	55–75
Lymphocytes × 10^3^	**0.63**	1.50–4.50
Monocytes × 10^3^	**1.77**	0.20–0.80
Eosinophils × 10^3^	**0.00**	0.05–0.50
Basophils × 10^3^	0.04	0.01–0.10
Platelets K/uL	**492.00**	150–450
Sodium (mEq/L)	135.70	135–145
Potassium (mEq/L)	4.85	3.6–5.2
Glucose (mg/dL)	87.80	70–100
LDH (U/L)	**466.64**	140–280
Ferritin (ng/mL)	**789.40**	12–300
D-dimer (ng/mL)	**4578.50**	< 500
IL-6 (pg/mL)	**42.20**	5–15
AST (U/L)	**68.00**	10–40
ALT (U/L)	**77.00**	7–56
pH	7.47	7.35–7.45
PaCO2 (mmHg)	**31.80**	35–45
PaO2 (mmHg)	**47.70**	80–100
HCO3 (mmol/L)	22.80	22–26
SaO2 (%)	**86.30**	95–100

Bolded values represent an out of range parameter

**Fig. 3:**
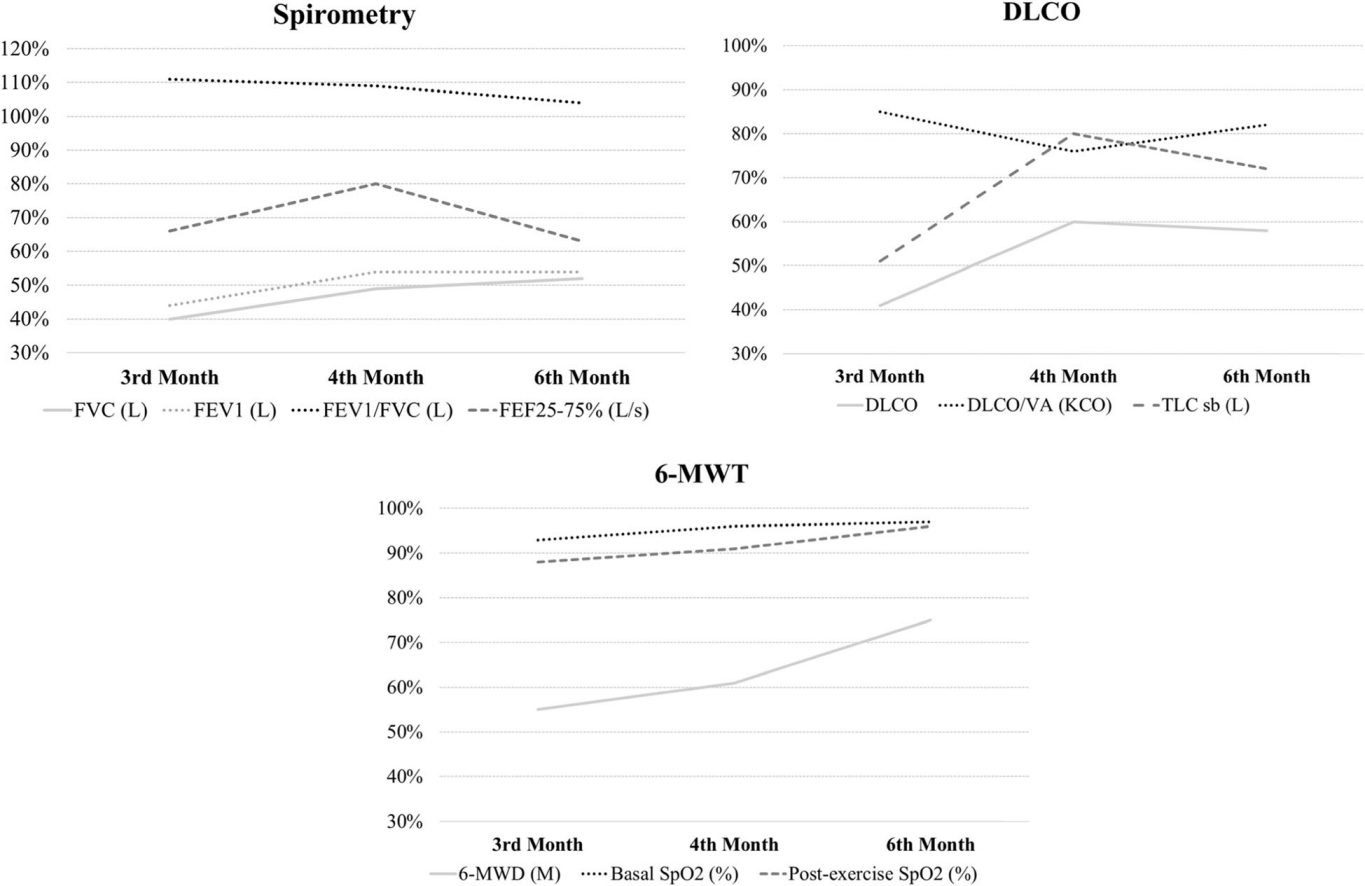
Spirometry, DLCO, and 6-min walk test at 3,4 and 6 months of follow-up. **Notes:** FVC, forced vital capacity; FEV1, forced expiratory volume in 1 s; FEF, forced expiratory flow; DLCO, diffusing capacity of carbon monoxide; VA; alveolar ventilation; KCO, carbon monoxide transfer coefficient; TLC, total lung capacity; 6-MWT, 6-m walk test; 6-MWD, 6-m walk distance; SpO2, oxygen saturation

Daily follow-ups were established to monitor for signs of clinical improvement. Two weeks later from the initial consultation the patient presented marked improvement in her symptoms, as well as in her CT scan (Fig. 1b), which prompted in discontinuation of the medications and the oxygen therapy. At one-month follow-up the patient presented a nearly complete resolution of the initial symptoms, with a partially restored capacity to perform her daily activities. At the 45th day a negative PCR test for the viral RNA was reported. However, spirometry and diffusion capacity for carbon monoxide (DLCO) at 3rd, 4th and 6th months showed results compatible with a restrictive pattern evidenced by reduced FVC, FEV1 and DLCO measurements with respect to predicted values (Fig. 3**,** Table 2), while the 6-min walk test showed a decreased walking distance. Radiographic changes persisted at the 6-month follow-up chest CT scan (Fig. 1c).

**Table 2:** Follow-up pulmonary function tests

Pulmonary Function Tests						
Month	3rd Month		4th Month		6th Month	
Parameter	Absolute Value	Predicted (%)	Absolute Value	Predicted (%)	Absolute Value	Predicted (%)
*Spirometry*						
FVC (L)	1.06	40%	1.31	49%	1.38	52%
FEV_1_ (L)	0.92	44%	1.12	54%	1.13	54%
FEV_1_/FVC (L)	0.87	111%	86.00	109%	81.70	104%
FEF25–75% (L/s)	1.40	66%	1.71	80%	1.33	63%
*DLCO*						
DLCO	8.30	41%	12.10	60%	11.70	58%
DLCO/VA (KCO)	4.52	85%	4.03	76%	4.32	82%
TLC sb (L)	2.00	51%	3.15	80%	2.86	72%
*6-MWT*						
6-MWD (M)	264	55%	294	61%	359	75%
Basal SpO2 (%)	93%	N/A	96%	N/A	97%	N/A
Post-exercise SpO2 (%)	88%	N/A	91%	N/A	96%	N/A

*FVC* forced vital capacity, *FEV1* forced expiratory volume in 1 s, *FEF* forced expiratory flow, *DLCO* diffusing capacity of carbon monoxide, *VA* alveolar ventilation, *KCO* carbon monoxide transfer coefficient, *TLC* total lung capacity, *6-MWT* 6-m walk test, *6-MWD* 6-m walk distance, *SpO2* oxygen saturation

## Discussion and conclusions

The medical literature is vast in tools designed to predict the probabilities of diseases, risk scores to predict prognosis and mortality, and several other methods which are aimed to assist the physician in daily clinical decision making to optimize patient outcomes and reduce adverse scenarios in an objective evidenced-based fashion [14]. Despite the fact that they may be prone to bias, several prediction models for diagnosis and prognosis of COVID-19 disease have been proposed with a good to excellent discriminative performance [15]. For instance, a retrospective study comparing 7 score systems developed for patients with community acquired pneumonia showed that A-DROP was the most reliable tool to stratify hospitalized patients by risk of death [16]. However, other publications have shown that even though scores for community acquired pneumonia provide a good source of therapeutic advice, they tend to underestimate viral infections [17, 18]. Other scoring systems, like the quick COVID Severity Index (qCSI), have been designed specifically to identify patients that may undergo respiratory deterioration in 24 of admission, whereas others like the Brescia-COVID Respiratory Severity Scale (BCRSS) algorithm may be easily accessible but not externally validated [19–21]. In the outpatient setting however, most of these scoring systems are often too technical and complex, requiring variables that are not easily accessible. This represents a huge challenge that is further complicated by the lack of a definitive and robust guideline.

The CDC and the WHO guidelines recommend high flow nasal cannula (HFNC) in patients that do not respond to conventional oxygen therapy. In a post hoc analysis including 310 patients with acute hypoxemic respiratory failure, HFNC resulted in a lower intubation rate compared to conventional oxygen therapy (35% versus 53%) and non-invasive ventilation (35% versus 58) [22]. Our patient presented with a SpO2 < 90% at 5-6 L/min at rest for 4 consecutive days with a low-flow nasal cannula, and despite this poor initial response, the patient had to be kept with conventional oxygen therapy due the unavailability of high-flow nasal devices in the outpatient setting. This unfortunate limitation inclined the management to a more prominent pharmacologic approach.

The Infectious Diseases Society of America (IDSA) guidelines recommend remdesivir over no antiviral drug, taking into consideration its in vitro activity against MERS-CoV, SARS-CoV 1 and 2, and a case series including 61 patients where remdesivir was prescribed in a compassionate basis with a 68% clinical improvement after a median follow-up of 18 days [23–25]. However, given the fact that the former publication was limited by the lack of a standard of care group to compare with, and upon the lack of additional supporting evidence, other guidelines like the WHO do not recommend any antiviral drugs unless patients are participating in a clinical trial. Our patient was not treated with any antiviral medication.

Even though there is insufficient data to recommend either for or against antiviral and immunomodulatory medication in patients with severe respiratory disease due to COVID-19, new evidence in the form of a large scale open-label study (RECOVERY trial) found a decrease in mortality with the use of corticosteroids [26]. In this study, the use of dexamethasone resulted in lower 28-day mortality in hospitalized patients with COVID-19 receiving some form of respiratory support (invasive mechanical ventilation or oxygen alone), compared to patients receiving usual care [26]. It should be noted however, that this benefit was exclusive to patients receiving respiratory support, leading to a change in management from what it was initially proposed by the CDC and the WHO that the use of systemic corticosteroids should be avoided in patients with no evidence of acute respiratory distress syndrome (ARDS). It is uncertain if this patient presented ARDS, however, upon the evidence of deteriorating and progressive disease, she was prescribed with a trial of 3 days of IV methylprednisolone, followed by 7 days of prednisone (40 mg PO). At the time the patient presented with severe COVID-19 disease, the decision was taken considering an observational study including 31 patients with mild COVID-19 disease, where no significant association between viral clearance time and corticosteroid treatment (hazard ratio [HR], 1.26; 95% CI, 0.58–2.74) was reported and indirect evidence in favor of corticosteroids for patients with severe respiratory disease secondary to H1N1 and SARS [27–30].

The use of immunomodulators as a strategy to block peripheral inflammation is not a new approach, but questions on its effectiveness to treat patients with COVID-19 remains open [31]. Currently, besides corticosteroids, no official guideline supports the routine use of immunomodulatory medications outside of the context of a clinical trial. Several agents are under active investigation as possible treatments, including interleukin-1 and 6 receptor blockers. In a small prospective cohort study the use of the interleukin-1 receptor blocker (anakinra) was associated with a reduced need for invasive mechanical ventilation in the ICU and mortality, although there are several limitations of this study that need to be addressed in clinical trials to further support these findings [32]. In the case of the interleukin-6 pathway inhibitor tocilizumab, early observational studies suggest a potential benefit in patients with COVID-19 and elevated pro-inflammatory cytokines (often referred to as “cytokine storm syndrome”) [33–35].

Our patient was started on colchicine. Colchicine can either inhibit microtubule assembly at intermediate doses or lead to depolymerization at high doses [36, 37]. These mechanisms are responsible for the expansive and complex pharmacodynamics of this drug, where its role as a modulator of cytokine signaling cascades and expression of extracellular mediators and receptors are perhaps of most interest in COVID-19 disease [36]. In a recent open-label, randomized clinical trial, the use of colchicine was associated with an improved time to clinical deterioration when compared to standard of care among hospitalized patients with COVID-19, however no difference was observed among biochemical endpoints between groups [38]. Several terms like “cytokine storm”, hyperinflammatory or secondary hemophagocytic lymphohistiocytosis-like syndrome have been extensively applied to describe a late and more severe course of COVID-19 infection, and as an inhibitor of NLRP3 inflammasomes and interleukin activation mitigator, colchicine may represent a viable alternative when tocilizumab is unavailable [39–42]. As a matter of fact, in the vascular tissue colchicine has shown to downregulate interleukin-1 and tumor necrosis factor alpha which are upstream mediators of IL-6, the main target of tocilizumab [43, 44]. Even though we cannot confirm it, our patient presented abnormally elevated ferritin, D-dimer, CRP, LDH, transaminases and IL-6, all of which are classic characteristics of this hyperimmune state, justifying to a certain extent the rational use of colchicine [45].

The inpatient management of COVID-19 patients has not yet been established with a high level of evidence, and certainly the care of severe disease in the outpatient setting is less clear. There are several limitations in this report regarding the clinical data and the management, but such limitations do also reflect the state of emergency and the chaos that resides in the health care systems of developing nations. For the ambulatory care of COVID-19 patients, several aspects of disease management may differ from current guidelines and basic requirements may represent a huge challenge to cover. Further research is needed to assist physicians in the daily clinical decision making, to optimize patient outcomes, and to reduce the probability of adverse scenarios of patients with COVID-19 managed in the ambulatory setting.

## Data Availability

Not applicable.

## Abbreviations

WHO: World Health Organization
CDC: Centers for Disease Control and Prevention
BCRSS: Brescia-COVID Respiratory Severity Scale
ARDS: Acute respiratory distress syndrome
HFNC: High-flow nasal cannula
